# Role of MHC-I Expression on Spinal Motoneuron Survival and Glial Reactions Following Ventral Root Crush in Mice

**DOI:** 10.3390/cells8050483

**Published:** 2019-05-21

**Authors:** Luciana Politti Cartarozzi, Matheus Perez, Frank Kirchhoff, Alexandre Leite Rodrigues de Oliveira

**Affiliations:** 1 Laboratory of Nerve Regeneration, University of Campinas–UNICAMP, Cidade Universitaria “Zeferino Vaz, Rua Monteiro Lobato, 255, 13083-970 Campinas, SP, Brazil; lucartarozzi@gmail.com; 2 School of Physical Education and Sport of Ribeirao Preto, University of Sao Paulo, Av. Bandeirantes, 3900, 14040-907 Ribeirão Preto, SP, Brazil; matheusperez@usp.br; 3 Molecular Physiology, Center for Integrative Physiology and Molecular Medicine (CIPMM), University of Saarland, Building 48, 66421 Homburg, Germany; Frank.Kirchhoff@uks.eu

**Keywords:** astrogliosis, PNS/CNS interface, microglial reaction, synaptic covering, β2m knockout mice

## Abstract

Lesions to the CNS/PNS interface are especially severe, leading to elevated neuronal degeneration. In the present work, we establish the ventral root crush model for mice, and demonstrate the potential of such an approach, by analyzing injury evoked motoneuron loss, changes of synaptic coverage and concomitant glial responses in β2-microglobulin knockout mice (β2m KO). Young adult (8–12 weeks old) C57BL/6J (WT) and β2m KO mice were submitted to a L4–L6 ventral roots crush. Neuronal survival revealed a time-dependent motoneuron-like cell loss, both in WT and β2m KO mice. Along with neuronal loss, astrogliosis increased in WT mice, which was not observed in β2m KO mice. Microglial responses were more pronounced during the acute phase after lesion and decreased over time, in WT and KO mice. At 7 days after lesion β2m KO mice showed stronger Iba-1^+^ cell reaction. The synaptic inputs were reduced over time, but in β2m KO, the synaptic loss was more prominent between 7 and 28 days after lesion. Taken together, the results herein demonstrate that ventral root crushing in mice provides robust data regarding neuronal loss and glial reaction. The retrograde reactions after injury were altered in the absence of functional MHC-I surface expression.

## 1. Introduction

Compression of spinal roots is a common clinical incident, and the root axotomy is directly related to the modification in neuronal function [1]. After trauma, there are axonal changes that indicate dysfunction and degeneration happening both proximal and distal to the lesion [2].

Distal to the lesion, the process, called Wallerian degeneration, is characterized by the deterioration of axon fragments [3]. Cell bodies of lesioned neurons undergo several morpho-functional changes that, together, are called chromatolysis. They comprise cell body hypertrophy, nucleus displacement, Nissl substance dissociation and expressive disturbance in the expression of structural molecules related to synaptic transmission [4].

The axotomy-induced retrograde synaptic responses are known as “synaptic stripping”, i.e., the extensive detachment of presynaptic terminals from perikarya and dendrites of axotomized motoneurons [5,6]. The plasticity of the nervous system ensures that structural and functional circuitry remodeling occurs after injury, in particular, with the detachment of the excitatory boutons that were in apposition with the lesioned neuron, which leads to a metabolic change and a shift from the synaptic transmission state to a recovery state [7,8,9]. In the acute phase after lesion, there is a significant reduction of synaptic inputs, even temporally ceasing the synaptic transmission [10,11].

The axotomy rapidly activates astrocytes and microglia in the vicinity of the affected synaptic terminals. After lesion, microglia and astrocytes quickly react through structural modifications of their cytoplasmic projections that get interposed between the axotomized-motoneuron membrane (post-synaptic membrane) and the retracting synaptic terminals [7,12,13,14]. Within a few days, axotomized motoneurons are surrounded by activated microglial cells which remain closely in apposition to the perikarya of injured neurons. The number of activated microglia in lesioned motor nuclei increases dramatically due to the proliferation that lasts for about two to four days post-axotomy [6].

Neurons express molecules such as the *major histocompatibility complex of class I* (MHC-I), a transmembrane complex [15] consisting of an α-chain associated with a light and obligatory extracellular chain, named β2-microglobulin (β2m) [16]. Classical MHC-I genes are well known for their role in the adaptive immune response and have their primary function related to binding peptides derived from intracellular proteolysis. The peptides that are processed intracellularly are loaded to the MHC-I molecules by the *Transporter associated with Antigen Processing 1* (TAP-1) protein for delivery to the cell surface; such peptides are recognized and identified by T-cytotoxic lymphocytes. Mice that are genetically deficient for β2m or TAP-1, lack stable cell surface expression of most MHC-I molecules [17].

MHC-I mRNA is expressed by neurons and glial cells in the olfactory system, cerebral cortex, striatum, hippocampus, and spinal cord, both during development and adulthood. MHC-I proteins are expressed on the surface of axons and dendrites and are also located pre- and post-synaptically [16,18]. In the healthy brain, MHC-I expression is regulated by neuronal activity [15,19] and its upregulation is related to critical periods where synaptic refinement occurs more substantially [15,20,21,22]. Therefore, MHC-I expression is directly related to synaptic re-organization of the Central Nervous System (CNS).

In the spinal cord, MHC-I and β2m are upregulated after peripheral lesion [23]. Furthermore, Oliveira, Thams, Lidman, Piehl, Hokfelt, Karre, Linda and Cullheim [9] observed more synaptic stripping after axotomy in β2m knockout (β2m KO) and in TAP-1 knockout (TAP1 KO) mice. Enhanced synapse detachment in β2m KO animals could be attributed to a preferential removal of inhibitory terminals, while excitatory terminals were removed to a similar extent in KO and wildtype (WT) mice; i.e., MHC-I molecules are important for a selective maintenance of inhibitory synaptic terminals after lesion.

We suggest that MHC-I signaling is used by neurons and glia to interact both in normal and pathological processes. MHC-I is expressed by astrocytes, microglia and at pre- and postsynaptic terminals to selectively maintain inhibitory terminals and glial processes that are putatively involved in the detachment of pre-synaptic buttons from the lesioned motoneuron cell body. Indeed, microglia and astrocytes reactivity after peripheral lesion are influenced by MHC-I modulation, also affecting the synaptic plasticity process and regenerative capacity [24,25]. However, the exact role of MHC-I in neuron-glia signaling and spatiotemporal events that outline this process is still elusive.

## 2. Materials and Methods

### 2.1. Mice and Experimental Groups

For this study, we used female C57BL/6J wild-type (WT) and B6.129P2B2mtm1Unc/J (β2m KO) mice. Mice homozygous for the β2m targeted mutation have little if any MHC-I protein expression on the cell surface.

Six to eight-week-old female mice were bred in-house, at the Laboratory of Nervous Regeneration, Institute of Biology, University of Campinas. Mice were kept in appropriate micro-isolators, under a 12 h light-dark cycle, with controlled temperature and humidity, with water and food ad libitum.

Protocols concerning the animal use and handling were approved by the Institutional Committee for Ethics in Animal Experimentation (Committee for Ethics in Animal Use—Institute of Biology—CEUA/IB/UNICAMP, Protocol number 3336-1) and were performed in accordance with the guidelines of the Brazilian College for Animal Experimentation. All experiments concerning animal experimentation.

### 2.2. Surgical Procedure for Ventral Root Crush (Figure 1)

Mice were anesthetized with xylazine (König, Argentina, 5 mg/kg) and ketamine (Fort Dodge, USA, 100 mg/kg. Bepanthen (Bayer, Germany) was applied in the eyes to prevent dryness. Mice were placed in a heated bed and the hair was removed from the back. In the absence of the toe-pinch withdraw, an incision parallel to the vertebral column was made at the thoracic level. Paravertebral muscles were removed to expose the lower thoracic and upper lumbar vertebrae. Laminectomy was performed to expose the spinal cord. After that, an incision was made in the dura-mater allowing to reach ventral roots correspondent to L4, L5 and L6 spinal segments that were crushed (3 times of 10 s) with number 4 forceps. After muscle and skin were sutured and mice were kept under controlled heating until completely recovered from anesthesia. A dose of Tramadol (Neoquimica, Brazil, 5 mg/Kg) was administered by gavage after surgery and for the following 3 days.

**Figure 2:**
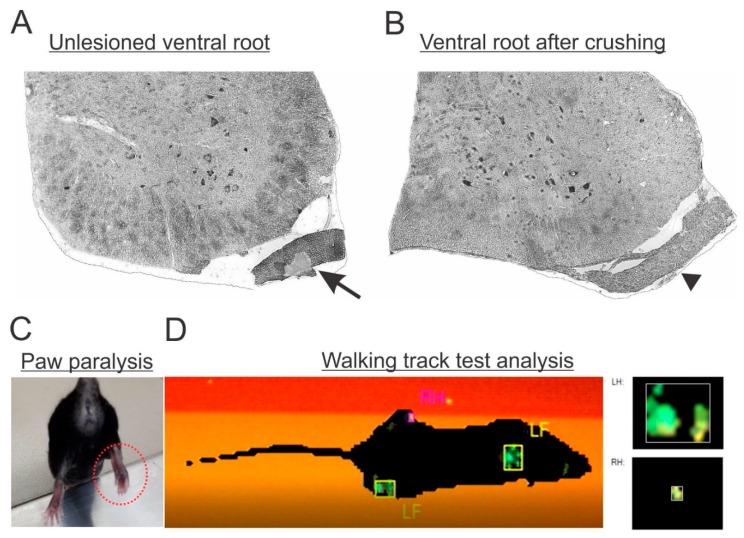
(**A**) Micrograph of a lumbar spinal cord transverse section showing the unlesioned ventral root morphology with intact axons (arrow). (**B**) Lesioned ventral root (arrowhead) with signs of ongoing Wallerian degeneration. (**C**) Paw paralysis behavior following L4, L5 and L6 ventral root lesion. Observe the adduction pattern of the toes combined with muscle atrophy (dotted red circle). (**D**) Gait analysis by the CatWalk system showing hindlimb loss of function ipsilateral to the lesion (RH).

To ensure that the lesion was successfully performed, in the following days after surgery, mice were behaviorally analyzed to check the correspondent paw paralysis (Figure 2C,D). Moreover, the morphology of fixed spinal cords was analyzed, to check the gross anatomical preservation of the nervous tissue after lesion, as well as to detect trace elements of degeneration on the axotomized roots (Figure 2A,B). Only mice fitting both criteria were used for further analysis. The success ratio of the surgery was estimated at 80%.

Each strain had three time-points of analysis after surgery, days after injury (dpi), constituting the following experimental groups: WT–7 dpi (n = 4), WT–14 dpi (n = 5), WT–28 dpi (n = 4), β2m KO–7 dpi (n = 4), β2m KO–14 dpi (n = 5), β2m KO–28 dpi (n = 4).

### 2.3. Perfusion

Mice were anesthetized with an overdose of xylazine and ketamine and submitted to thoracotomy followed by transcardiac perfusion with PBS (0,1M Sodium Phosphate Buffer, PB, with 0.9% NaCl; pH 7.38). Afterwards, mice designated for the analysis of motoneuron survival and immunohistochemistry were perfused with a fixative solution (4% Formaldehyde in 0.1 M PB). After fixation, lumbar intumescences were dissected out and immersed in the same fixative solution overnight, at 4 °C, washed 3 times with 0.1 M PB, immersed in sucrose solutions (10%, 20% and 30%, 12 h each), finally soaked in Tissue-Tek, frozen in n-Hexane at controlled temperature (−32 °C to −35 °C) and stored at −20 °C.

### 2.4. Immunohistochemistry

Frozen 12 μm–thick serial sections were obtained using a cryostat (Microm, HM525), transferred to a gelatin-covered microscopic slide and stored at −20 °C until use.

For immunohistochemistry, microscopic slides were left at room temperature and the sections were outlined by a hydrophobic pen. Slides were transferred to a humid and light-protected chamber. Sections were immersed in 0.01 M PB (3 times, 5 min each), dried and incubated with 150 μL of blocking solution (3% Fetal Bovine Serum in 0.1 M PB) for 45 min. Subsequently, the different primary antibodies (Table 1) were diluted in incubation solution (1.5% Fetal Bovine Serum and 0.2% Tween in 0.1 M PB) and sections were incubated for 4 h or overnight.

After first incubation, slice sections were washed with 0.01 M PB and incubated with the respective secondary antibodies (cy2 anti-rabbit, cy3 anti-mouse or cy3 anti-rat; Jackson Immunoresearch, 1:500) for 45 min. As a nuclear marker, DAPI (4’,6-diamidino-2-phenylindole, 1:1000 in 0.1 M) was used. Sections were again washed with 0.01 M PB and mounted with coverslips using as medium glycerin/PB (3:1).

Immunostainings were observed with an epifluorescence microscope (Leica DMB5500, Leica, Wetzlar, Germany) and documented with a digital camera (Leica DFC 345 FX), using specific filters according to the secondary antibodies or DAPI.

For quantification, three representative images from each animal of the respective experimental group were selected. The integrated density of pixels, representing protein immunolabeling, was measured in the lateral motor nucleus at anterior horn from the ipsi- and contralateral sides of the spinal cord, according to Oliveira, Thams, Lidman, Piehl, Hokfelt, Karre, Linda and Cullheim [9], using Image J software (1.51j8 version, National Institutes of Health, NIH, USA). As illustrated in Supplementary Figure S1, for GFAP and IBA-1, the whole area of the picture was quantified; for anti-synaptophysin immunolabeling, eight small areas around each motoneuron were measured (Supplementary Figure S2).

Integrated density of pixels was acquired for each animal and then the mean ± standard error of each experimental group was calculated.

### 2.5. Putative Motoneuron Cell Survival

For motoneuron-like cells counting, tissue specimens were processed just as described for immunohistochemistry, until sectioning and storage of the microscopic slides. Transversal sections from the lumbar intumescence were stained in 0.5% Toluidine Blue (Synth, Diadema, Brazil) for 1 min, rinsed in water, dehydrated, diaphanized and mounted with Entellan (Merck, Darmstadt, Germany) and coverslip.

Following, putative motoneurons from the lateral motor nucleus of the anterior horn of the spinal segments L4, L5 and L6 were counted in the ipsi- and contralateral sides every four slides from the specimen. In order to correct double counting, Abercrombie’s formula was used [26]:

N = n × t/(t + d)

“N” is the corrected number of neurons, “n” is the number of counted cells, “t” is the section thickness and “d” is the average diameter of the neurons. Once the neuron size difference affects the corrected number of neurons, the “d” value was calculated for each experimental group (ipsi- and contralateral).

### 2.6. Statistical Analysis

The data are presented as the mean ± SEM and analyzed using one-way ANOVA followed by Bonferroni post-hoc test, for multiple comparisons or two-tailed t-test; at *p* < 0.05 (*), *p* < 0.01 (**), and *p* < 0.001 (***).

## 3. Results

### 3.1. Time-Course of Motoneuron Survival, Glial Reaction, and Synaptic Covering Following VRC in C57BL/6J Mice

Neuronal survival rate was evaluated 7, 14 and 28 days after ventral root crush (VRC) by the counting of large α-motoneurons-like cells present in the ipsilateral lamina IX and compared to the contralateral side (ipsi/contralateral ratio). No statistical differences between the contralateral sides from C57BL/6J (WT) mice at the different time points were observed (mean ± standard error for different experimental groups: 7 dpi, 6.65 ± 0.17; 14 dpi, 6.71 ± 0.44; 28 dpi 7.8 ± 0.24).

**Figure 3:**
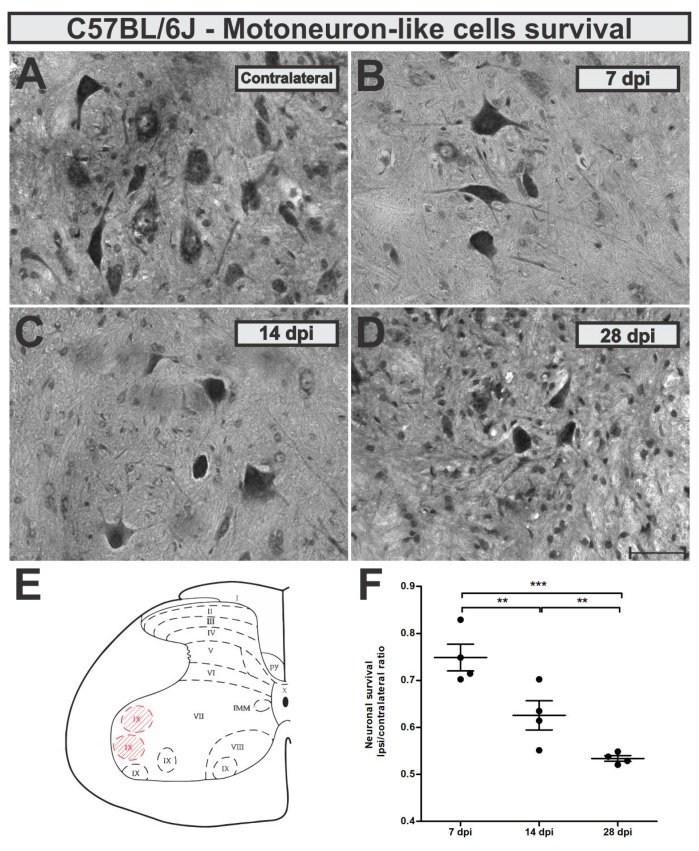
Time-course of motoneuron-like cell survival in the wild type (WT) mice (**A**) contralateral - unlesioned, (**B**) 7 days after injury (dpi), (**C**) 14 dpi and (**D**) 28 dpi. (**E**) Schematic spinal cord transverse section showing the laminae of Rexed distribution. Lamina IX is highlighted in red and represents the area where cell counting, and immunohistochemistry analysis were performed. (**F**) Neuronal survival at the time points after ventral root crush (ipsi/contralateral ratio); *p* < 0.01 (**), and *p* < 0.001 (***). Scale bar = 50 µm.

Seven days after VRC, C57BL/6J mice showed already 25% of neuron loss which increased to 38 and 47% 14 and 28 days later, respectively (Figure 3). Loss of motoneuron-like cells in the lateral motor nucleus from the lumbar intumescence was statistically higher 14 and 28 days after VRC when compared to 7 dpi (*p* < 0.0001, F _2,11_ = 25.83, Bonferroni post-test). 

Description of calculated motoneuron survival means ± standard errors from C57BL/6J experimental groups: 7 dpi = 0.64 ± 0.02; 14 dpi = 0.55 ± 0.02; 28 dpi = 0.49 ± 0.02.

**Figure 4:**
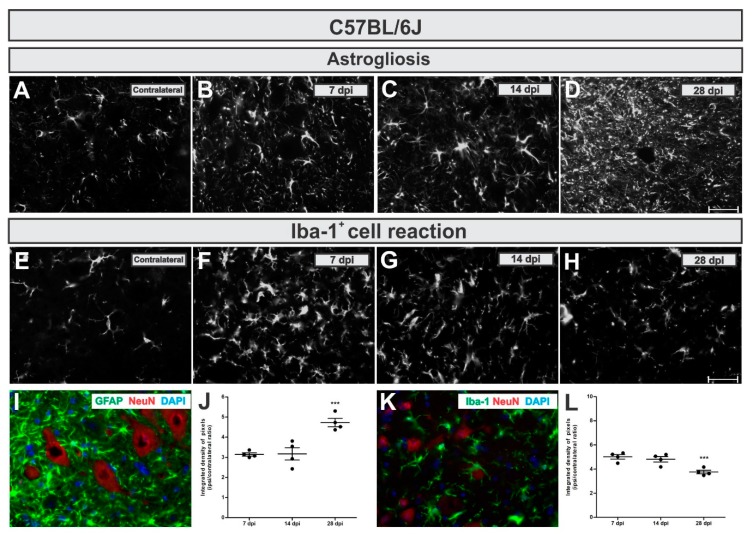
Time-course of astrogliosis (**A** to **D** and **I**) and Iba-1^+^ cell reaction (**E** to **H** and **L**) in the lumbar spinal cord contralateral side and 7, 14 and 28 dpi post ventral root injury in WT mice. Note that astrogliosis increases over time. Contrarily, the Iba-1^+^ cell reaction is more intense in the acute phase after injury. **I** and **K** illustrate the glial reaction in the ipsilateral side nearby axotomized motoneuron-like cells, positive to NeuN (in red). (**J** and **L**) show the comparative quantification among time points post injury (ipsi/contralateral ratio); *p* < 0.001 (***). Scale bar = 50 µm.

To analyze astroglial changes in the lesioned neuronal microenvironment we used immunohistochemical labeling of glial fibrillary acid protein (GFAP)—an astrocytic intermediary filament marker. In WT mice, astrogliosis increased in a time-dependent manner, reaching five-fold upregulation by 28 days after injury (Figure 4A,D,I,J). Comparing the time points, increased astrogliosis was statistically higher 28 dpi when compared to 7 and 14 dpi (*p* = 0.0005, F2,10 = 18.17, Bonferroni post-test).

The calculated IL/CL integrated density of pixels ratio mean ± standard error from GFAP immunoreactivity in C57BL/6J - WT experimental groups provided the following results: 7 dpi = 3.14 ± 0.1; 14 dpi = 3.25 ± 0.4; 28 dpi = 4.72 ± 0.2.

To analyze microglia and macrophages changes surrounding lesioned motoneurons, we used immunolabelling of Iba-1 (Ionized calcium binding adaptor molecule–a microglia/macrophage calcium-binding marker). It is important to point out that VRC is a lesion that results in the blood brain barrier disruption and Iba-1 also labels macrophages. Thus, Iba-1 positive labelling nearby motoneurons does not exclude macrophages derived from circulating monocytes.

In C57BL/6J mice, on the contrary to what was detected for astrogliosis, the Iba-1^+^ cells reaction was more intense in the acute phase after lesion (7 days), becoming reduced by 26% at day 28 (Figure 4E,H,K,L). The reduction in the microglial reaction in the lateral motor nucleus of WT mice at 28 dpi was significantly different compared to 7 and 14 dpi (*p* = 0.0031, F2,10 = 10.84, Bonferroni post-test).

Calculating IL/CL integrated density of pixels ratio mean ± standard error from Iba-1 immunoreactivity in C57BL/6J experimental groups led to the following data: 7 dpi = 5.02 ± 0.2; 14 dpi = 4.68 ± 0.2; 28 dpi = 3.76 ± 0.2.

To analyze synaptic changes after VRC, we used an anti-synaptophysin antibody and assessed pre-synaptic terminals in apposition to the axotomized neurons as described in Methods section.

**Figure 5:**
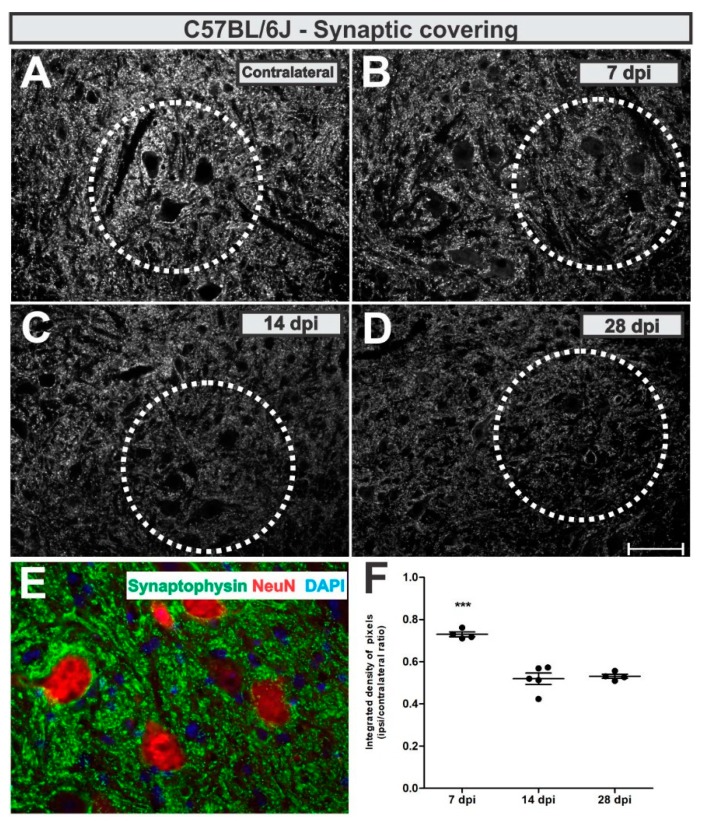
Synaptic covering in WT mice. (**A**) representative contralateral image, (**B**) 7 dpi, (**C**) 14 dpi and (**D**) 28 dpi. (**E**) illustrates the synaptic inputs (in green) to ipsilateral motoneuron-like cells, labelled with NeuN (in red). (**F**) Comparative quantification among time points (ipsi/contralateral ratio); *p* < 0.001 (***). Scale bar = 50 µm.

Synaptic inputs were reduced by 27% adjacent to the putative axotomized motoneurons at day 7 after lesion, which was further reduced to 47% (*p* < 0.0001, F2,10 = 34.21, Bonferroni post-test), both 14- and 28-days post-lesion (Figure 5).

The calculated IL/CL integrated density of pixels ratio mean ± standard error from synaptophysin immunoreactivity in C57BL/6J - WT experimental groups resulted in the following values: 7 dpi = 0.73 ± 0.05; 14 dpi = 0.52 ± 0.03; 28 dpi = 0.53 ± 0.05.

### 3.2. Enhanced Microglial Reaction and Synaptic Stripping in β2mKO Mice after VRC

**Figure 6:**
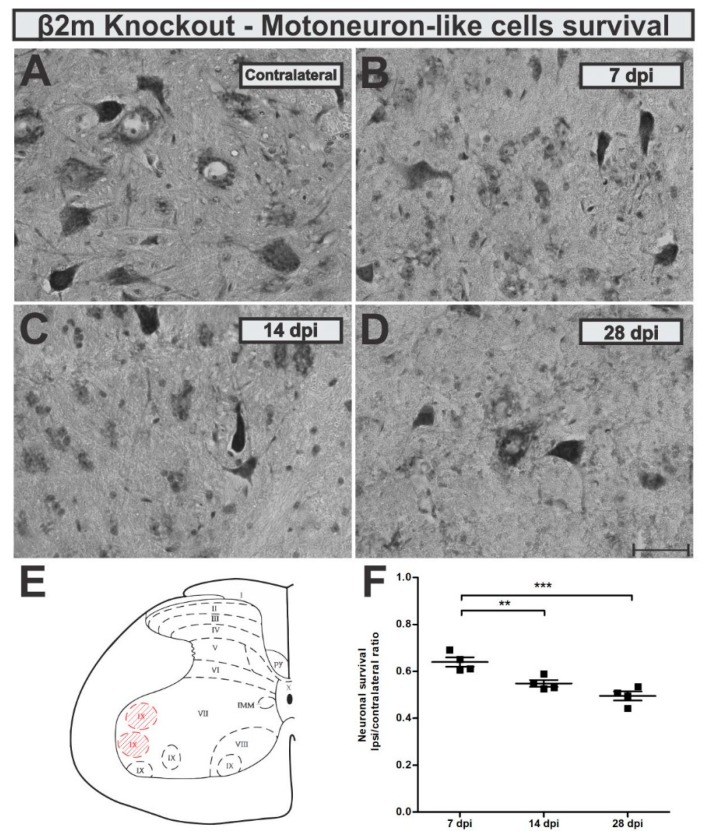
Time-course of motoneuron-like cell survival in the β2m knockout mice (**A**) contralateral - unlesioned, (**B**) 7 dpi, (**C**) 14 dpi and (**D**) 28 dpi. (**E**) Schematic spinal cord transverse section showing the laminae of Rexed distribution. Lamina IX is highlighted in red and represents the area where cell counting, and immunohistochemistry analysis were performed. (**F**) Neuronal survival at the time points after ventral root crush (ipsi/contralateral ratio); *p* < 0.01 (**), and *p* < 0.001 (***). Scale bar = 50 µm.

After establishing the VRC lesion model in wildtype mice, we used β2m KO mice to test glial responses and neuronal survival in a condition with impaired antigen presentation and altered neuron-glia communication. First, we monitored motoneuron-like cells survival rate at 7, 14 and 28 days after VRC. Seven days after VRC, the KO mice showed 36% of neuron loss which increased to 45 and 51% fourteen and twenty-eight days after lesion, respectively (Figure 6). Statistically, the motoneuron-like cells loss was significantly higher 28 days after VRC when compared to 7 dpi (*p* = 0.0003, F 2,10 = 20.66, Bonferroni post-test). 

**Figure 7:**
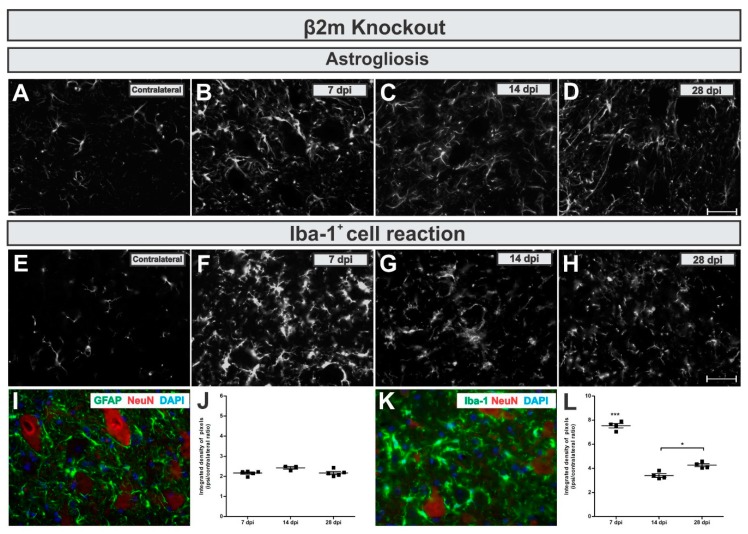
Time-course of astrogliosis (**A** to **D** and **I**) and Iba-1^+^ cell reaction (**E** to **H** and **L**) in the lumbar spinal cord contralateral side and 7, 14 and 28 dpi post ventral root injury in β2m knockout mice is kept constant over time and microglial reaction is more intense in the acute phase after injury. **I** and **K** illustrate the glial reaction in the ipsilateral side nearby axotomized motoneuron-like cells, positive to NeuN (in red). (**J** and **L**) show the comparative quantification among time points post injury (ipsi/contralateral ratio); *p* < 0.05 (*) and *p* < 0.001 (***). Scale bar = 50 µm.

When looking at the reactive astrogliosis in β2mKO mice, we observed that GFAP was upregulated in the IL side of about 2-fold related to the CL (Figure 7A,D,I,J), but no changes among the time points were detected (*p* = 0.511, F 2,11 = 0.7138, Bonferroni post-test).

By calculating the IL/CL integrated density of pixels ratio mean ± standard error from GFAP immunoreactivity in β2m KO groups the following data was obtained: 7 dpi = 2.17 ± 0.2; 14 dpi = 2.5 ± 0.2; 28 dpi = 2.16 ± 0.2.

The Iba-1^+^ cells reaction in β2mKO mice showed a strong transient upregulation. The Iba-1 immunolabel reached a seven-fold increase at 7 days after VRC, which was then reduced to four-fold upregulation day 28 (Figure 7E,H,K,L). When statistically compared to other time points, the increased reaction in the lateral motor nucleus from the lumbar intumescence was statistically higher 7 days after VRC when compared to 7 and 14 dpi (*p* < 0.0001, F 2,10 = 106.1, Bonferroni post-test).

Description of the calculated IL/CL integrated density of pixels ratio mean ± standard error from Iba-1 immunoreactivity in 7dpi in β2m KO groups: 7 dpi = 7.53 ± 0.2; 14 dpi = 3.40 ± 0.2; 28 dpi = 4.16 ± 0.2. 

**Figure 8:**
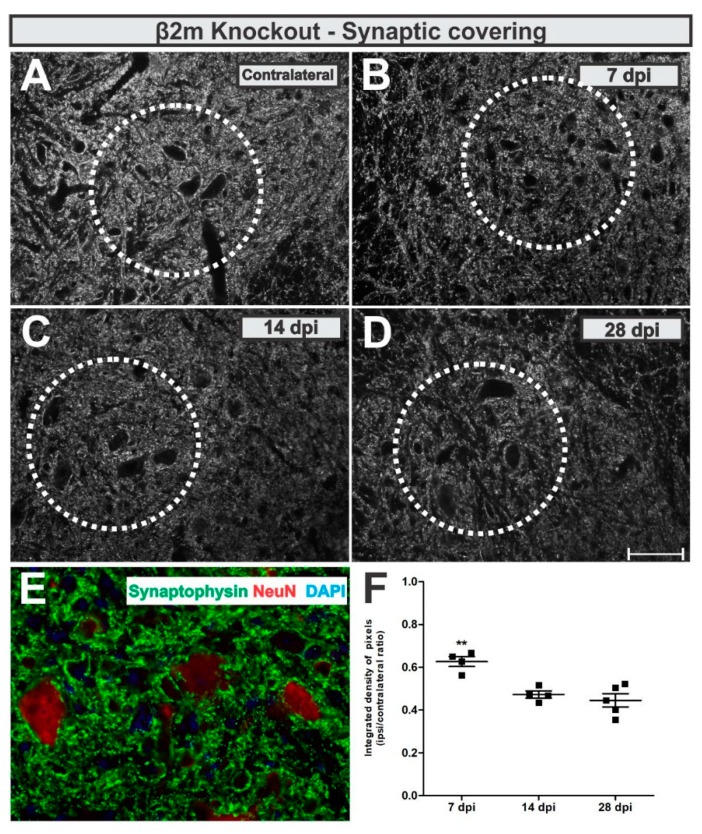
Synaptic covering in β2m knockout mice. (**A**) representative contralateral image, (**B**) 7 dpi, (**C**) 14 dpi and (**D**) 28 dpi. (**E**) illustrates the synaptic inputs (in green) to ipsilateral motoneuron-like cells, labelled with NeuN (in red). (**F**) Comparative quantification among time points (ipsi/contralateral ratio); *p* < 0.01 (**). Scale bar = 50 µm.

In β2m KO mice, the analysis of the anti-synaptophysin immunolabeling around lesioned motoneuron-like cells showed that the synaptic coverage was reduced by 48% at day 7 after lesion, reaching 56% reduction at day 28 (Figure 8). When statistically compared, the synaptic coverage was 29% lower 28 days after VRC when compared to 7 dpi (*p* = 0.0013, F 2,10 = 13.87, Bonferroni post-test).

IL/CL integrated density of pixels ratio means ± standard errors from synaptophysin immunoreactivity β2m KO groups led to the following data: 7 dpi = 0.62 ± 0.05; 14 dpi = 0.47 ± 0.03; 28 dpi = 0.44 ± 0.05.

### 3.3. Comparison between C57BL/6J and B2m KO Responses to Injury

**Figure 9:**
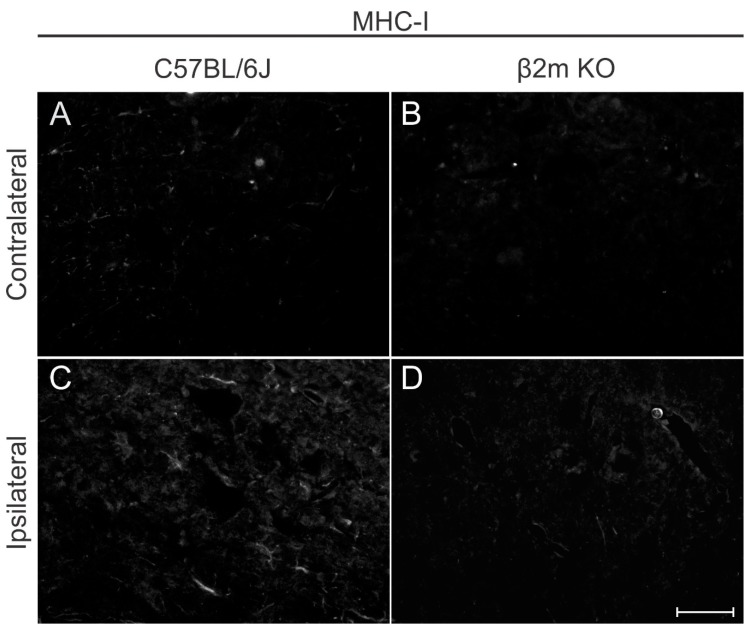
Major histocompatibility complex of class I (MHC-I) expression in C57BL/6J (**A**,**C**) and β2mKO mice (**B**,**D**) 7 days after VRC. Note that contralateral labeling is almost absent in C57BL/6J showing upregulation after injury. β2mKO mice showed weak staining both in the contra and ipsilateral sides. Scale bar = 25 µm.

A qualitative MHC-I immunolabeling was performed 7 days post lesion in both C57BL/6J and β2m KO mice (Figure 9) to show that there is a low basal labelling in WT contralateral side and an upregulation in MHC-I labeling after lesion. In β2m KO mice the labelling is almost absent and the upregulation after lesion cannot be depicted.

**Figure 10:**
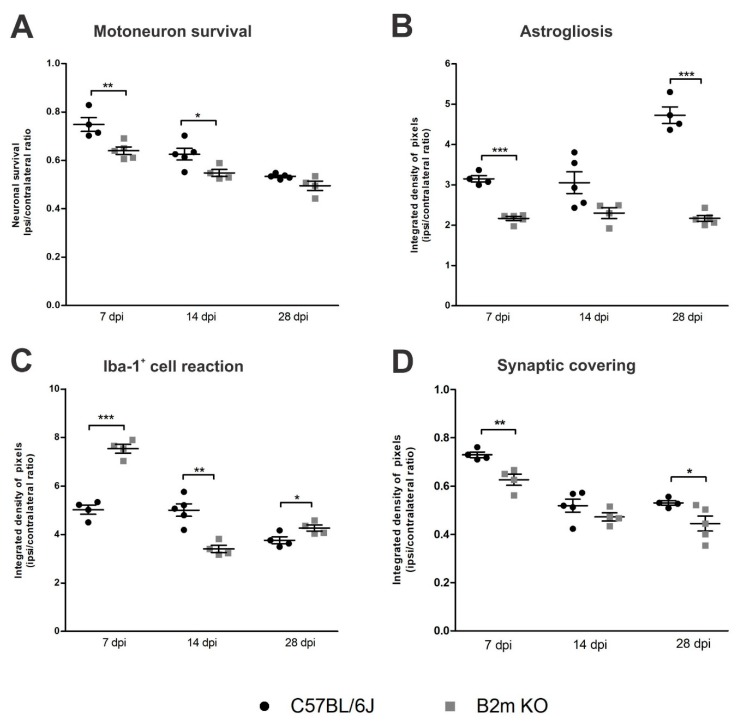
Comparative time course analysis of motoneuron-like cell survival (**A**), astrogliosis (**B**), Iba-1^+^ cell reaction (**C**) and synaptic covering (**D**) in WT and β2m KO mice. Note that the progressive loss of motoneuron-like cells is equivalent in both strains, despite astrogliosis in β2m KO that did not show increasing over time. On the other hand, the Iba-1^+^ cells reaction was more intense in β2m KO mice in the acute phase after VRC, which was coincident to a more pronounced synaptic detachment in the same strain, a difference that was detected also 28 dpi. Graphs show the comparative quantification among time points (ipsi/contralateral ratios); Significance levels: * *p* < 0.5, ** *p* < 0.01, *** *p* < 0.001.

Concerning putative motoneuron survival, when comparing the results from C57BL/6J and β2mKO mice (Figure 10), for each time point, the motoneuron loss at day 7 post-injury was sharply higher in β2m KO compared to the WT (*p* = 0.0094, R^2^ = 0.6425, t-test), pointing out that there is an increased neuronal susceptibility in this phase in the absence of MHC-I.

Unlesioned spinal cords of C57BL/6J and β2mKO mice were labeled with GFAP and Iba-1 antibodies and no differences between the strains could be depicted (Supplementary Figure S3).

The reactive astrogliosis (Figure 10) was significantly higher (*p* < 0.0001, R^2^ = 0.95, t-test) in β2mWT mice 7 and 28 dpi, when compared to β2m KO. On the other hand, reaction of Iba-1^+^ cells at 7 dpi was higher in β2m KO compared to C57BL/6J (*p* < 0.0001, R^2^ = 0.9395, t-test) and, at 14 dpi this reaction was 27% reduced in the β2m KO when compared to the WT (*p* = 0.0015, R^2^ = 0.7824, t-test) pointing out that following the exacerbate Iba-1^+^ cells reaction in the β2mKO at day 7 post-injury, there was an intense reduction by the 14th day in the absence of MHC I, when compared to WT.

The comparative analysis from WT and β2m KO synaptic covering after VRC showed that the synaptic loss was 16% higher in β2m KO mice 7 and 28 dpi when compared to WT (*p* < 0.007, R^2^ = 0.783, t-test).

## 4. Discussion

We show the time course of neuronal degeneration, synapse retraction, and glial reaction after ventral root crushing in C57BL/6J - WT mice and compared to β2mKO mice. 

In the first week after lesion, 25% of putative motoneuron loss was already detected, increasing to 38% in the second week and reaching 47% in the fourth week after VRC, showing similarities with data already published regarding VRC in rats, which presented motoneuron loss up to 51%, 28 days after lesion [27].

As expected, astrogliosis increased in the ipsilateral side after lesion to the CNS/PNS interface [28,29], reaching in the mouse 3-fold upregulation between 7 to 14 days, increasing in a time-dependent manner and reaching 5-fold upregulation by 28 days after injury. On the other hand, the Iba-1^+^ cells reaction was also induced by VRC, being more intense in the acute phase after lesion (7 days) and becoming reduced by 26% at day 28.

Concerning the synaptic coverage, synaptic inputs were reduced by 27% nearby axotomized motoneurons at day seven after lesion, and such coverage was reduced to 47% until 28 days post-injury. These data corroborate with previous results from our group [27,28,29] Thus, the results described so far demonstrate that ventral root crushing in mice provides robust data regarding neuronal loss and glial reaction. This allows studies with transgenic strains and/or therapeutic approaches that may, in turn, unveil strategies to improve motor recovery after proximal lesions.

In this context, we performed the same time course analysis in β2m KO mice. It is already known that MHC-I influences the interaction between pre- and post-synaptic neurons as well as between neurons and glial cells. Also, this molecule is upregulated both in the CNS and PNS as a response to axotomy [23,24,30]. The lack of MHC-I showed decreased motoneuron axonal regeneration after peripheral lesion [9]. On the other hand, the enhanced neuronal MHC-I expression has a beneficial effect following spinal cord injury, leading to the significantly better recovery of locomotor abilities [31].

Following VRC, the absence of MHC-I plays an important role, especially acutely post-injury (7 dpi). In this case, in β2mKO mice, the motoneuron-like cell loss was 33% higher at this time point, astrogliosis was decreased and the Iba-1^+^ cells reaction was more intense when compared to WT mice.

Bombeiro, et al. [32] showed in vitro a reduction in GFAP-labelling and hypertrophic astrocytes following β2m knock down by using siRNA (small interfering RNA). In a co-culture system, the reduced expression of GFAP in knocked down astrocytes was related to maintenance of synaptophysin immunostaining in neurons. Similarly, seven days after VRC, we observed 31% less GFAP immunoreactivity in the lateral motor nucleus at lamina IX from β2m KO mice when compared to WT. This reduced reactive astrogliosis was even more accentuated 28 days post-injury, 54% lower in β2m KO when compared to C57BL/6J-WT. 

It is important to point out that astrocytes play an essential role in neurotransmitter reuptake from the synaptic space and this feature is especially important under deleterious stimuli, such as a lesion. The loss of synaptic inputs from the lesioned motoneuron cell body occurs preferentially to excitatory inputs, subjecting the cells mostly to an inhibitory influence during repair [33]. In the lack of MHC-I, the selective maintenance of inhibitory synaptic terminals after axotomy is altered and a larger number of inhibitory terminals are removed [9].

Chen, et al. [34] pointed out that, in the adult brain, microglia-mediated synaptic stripping of pre-synaptic terminals is neuroprotective and the activated microglia in close apposition to neurons displaced GABAergic synapses. Chen, Jalabi, Hu, Park, Gale, Kidd, Bernatowicz, Gossman, Chen, Dutta and Trapp [34] reported that, in the adult brain, microglia-mediated synaptic stripping of pre-synaptic terminals is neuroprotective and the activated microglia in close apposition to neurons displaced GABAergic synapses. In this scenario, we propose that the higher number of inhibitory terminals retracting in β2m KO mice after lesion [9] could be due to physical displacement by microglial processes. Indeed, seven days after VRC, the Iba-1^+^ cells reactivity detected by anti-Iba-1 immunostaining showed that β2m KO had 33% more reactive cells when compared to WT. Even though Iba-1 positive cells in this scenario include microglia and macrophages derived from circulating monocytes, it is plausible to assume that the microglial reaction is also enhanced in this situation. Thus, we hypothesize that the higher synaptic loss in β2m knockout mice, is due to the enhanced microglial activation, which is actively involved in the detaching of synaptic inputs.

Liddelow, et al. [35] showed that astrocytes with a harmful profile (defined by the authors as A1 astrocytes) are induced by factors secreted by activated microglia (namely: IL-1α, TNF and C1q). A1 astrocytes lose their normal astrocytic function, such as synaptic maintenance, and can also be induced by axotomy and neurodegenerative diseases. In a model of optic nerve crush, a fast generation of A1 astrocytes in parallel to the death of retinal ganglion cells was detected. Testing the deleterious effects of A1-like astrocytes on several sorts of neuronal types, it was detected that 20% of spinal motoneurons remained viable after submitted to A1-like astrocytes conditioned medium, while other cells, like retinal ganglion cells, did not survive at all. Moreover, γ-motoneurons were not susceptible to A1 astrocyte toxicity [35].

Reactive astrogliosis or microglial reaction to injury or neurodegenerative process did not occur in isolation but as part of a multicellular response [36]. So, in response to injury, a glial response occurs, constituting both micro- and astrogliosis and leading to a local innate inflammatory reaction in the vicinity of the axotomized nerve cells. In a model of ventral root avulsion, for example, after approximately 1 week there is a phase of microglia activation paralleled by up-regulation of astrogliosis and lymphocyte infiltration, coinciding with the beginning of the death of axotomized cells, that are about 50% gone by the third week [37]. As, after a traumatic lesion, the microenvironment changes dictate the destiny of axotomized motoneurons and the maintenance of synaptic connectivity through glial reaction, the lack of a functional MHC-I can modulate the inflammation accentuating glial reaction towards a harmful way causing ultimately a more pronounced synaptic detachment in the lesion as described here.

## Figures and Tables

**Figure 1:**
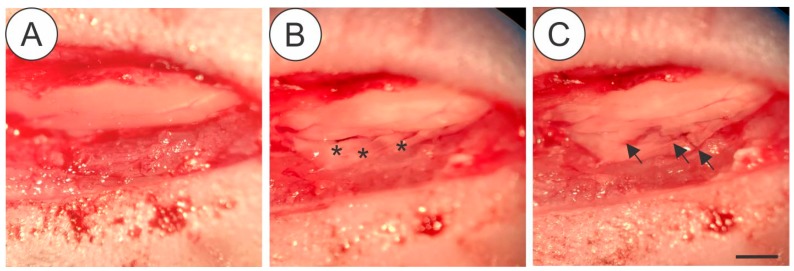
Details of the surgical procedure for ventral root crush in mice showing the exposed spinal cord with intact dura-mater after laminectomy (**A**), the L4, L5 and L5 ventral roots (asterisk) isolated after dura-mater longitudinal incision (**B**) Crushed roots (**C**-arrows), where it is possible to depict the persistence of the connective tissue at the injury site. Scale bar = 1 mm.

**Table 1:** Detailed description of the primary antibodies used.

Antibody	Host	Company	Code	Dilution
Anti-Iba-1	Rabbit	Wako	019-19741	1:750
Anti-GFAP	Rabbit	Abcam	ab7260	1:1500
Anti-Synaptophysin	Rabbit	Novus Biologicals	NBP2-25170	1:1000
Anti-MHC-I	Rat	BMA Biomedicals	T2105	1:100
Anti-NeuN	Mouse	Millipore	MAB377	1:500

## Supplementary Materials

The following are available online at https://www.mdpi.com/2073-4409/8/5/483/s1.
