# Supplementary material for: Role of MHC-I Expression on Spinal Motoneuron Survival and Glial Reactions Following Ventral Root Crush in Mice
Source: Cells. 2019 May 21;8(5):483. doi: 10.3390/cells8050483 (PMC6563038; doi:10.3390/cells8050483)
Supplement: Supplementary file 1 [file cells-08-00483-s001.pdf]

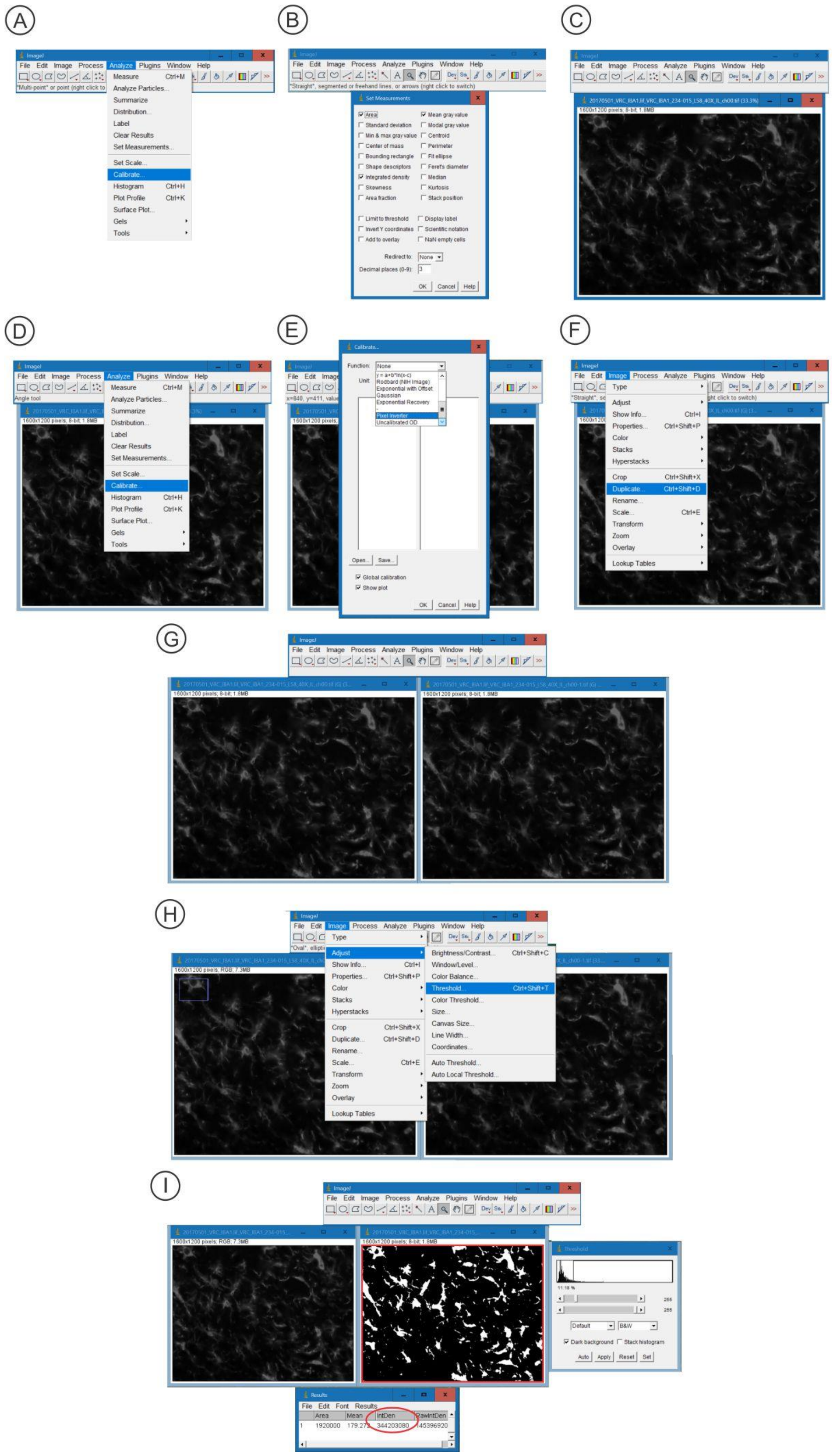

Figure S1 – Step by step method for obtaining the integrated density of pixels quantification of GFAP and Iba-1 immunolabeling using ImageJ. Firstly, start ImageJ and, in the Analyze tab, select Set Measurements (**A**) and choose Area and Integrated Density (**B**). Open an 8-bit image (**C**) and, in the Analyze tab, select Calibrate (**D**). Scroll the menu and choose Pixel Inverter; click on Global Calibration (**E**). After calibration, duplicate the image using Image >> Duplicate (**F**). Select one of the

images (**G**), and in the Imaging menu select Adjust >> Threshold (**H**). This function turns your image into a black-and-white pattern in which the labeling intensity can be changed with the scroll bar. Use the duplicate image to compare the exact pattern of the labeling in the Threshold window and click Apply (**I**). After that, press Ctrl + M for measuring the total area of the image. The integrated density value (IntDen) must be transferred to an Excel spreadsheet. For each animal, 3 images were measured, and the mean integrated density of pixels was calculated.

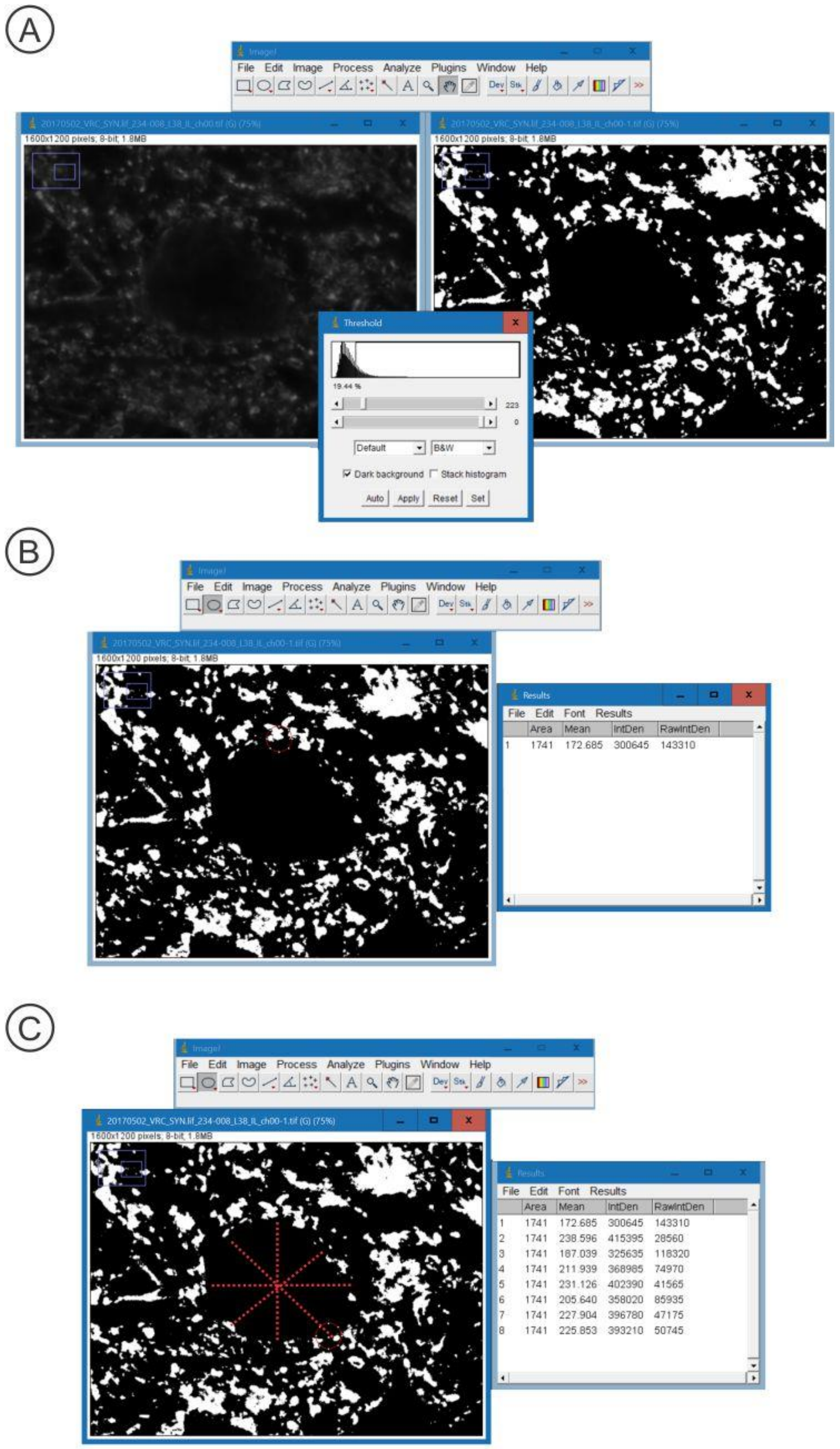

Figure S2 – Step by step method, using ImageJ, for obtaining the integrated density of pixels quantification of Synaptophysin immunolabeling. Follow the same steps described in Supplementary Figure 1 until Threshold. From that, after motoneuron identification and Threshold setting (**A**), a

small circle with constant diameter was drawn within the image and positioned on the motoneuron cell membrane and the measurement acquired (Ctrl + M) (**B**). Measurements were acquired in 8 equidistant points of motoneuron cell membrane (**C**). For each image, two motoneurons were analysed. Three images were quantified per animal and the mean integrated density of pixels mean was then calculated.

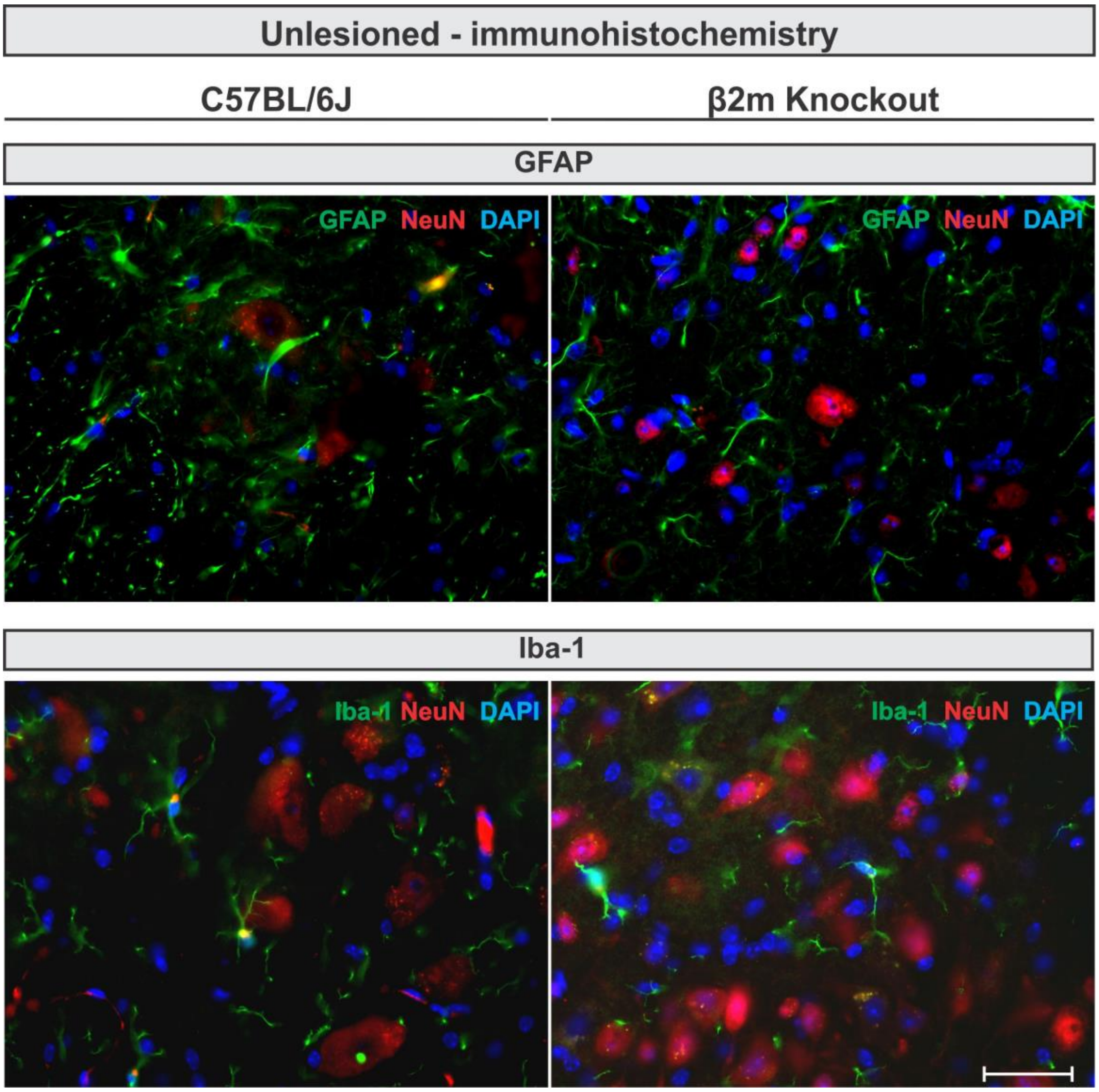

Figure S3 – Anti-GFAP and anti-Iba-1 in unlesioned C57BL/6J and β2m knockout mice spinal cords, specifically at lamina IX, around putative ventrolateral motoneurons (labeled with NeuN, in red) showing no differences in glial labeling pattern among the strains.
